# Severe, short-term sleep restriction reduces gut microbiota community richness but does not alter intestinal permeability in healthy young men

**DOI:** 10.1038/s41598-023-27463-0

**Published:** 2023-01-05

**Authors:** J. Philip Karl, Claire C. Whitney, Marques A. Wilson, Heather S. Fagnant, Patrick N. Radcliffe, Nabarun Chakraborty, Ross Campbell, Allison Hoke, Aarti Gautam, Rasha Hammamieh, Tracey J. Smith

**Affiliations:** 1 Military Nutrition Division, U.S. Army Research Institute of Environmental Medicine, Natick, MA USA; 2 Oak Ridge Institute of Science and Education, Oak Ridge, TN USA; 3 Medical Readiness Systems Biology, CMPN, Walter Reed Army Institute of Research, Silver Spring, MD USA; 4 Geneva Foundation, Walter Reed Army Institute of Research, Silver Spring, MD USA

**Keywords:** Applied microbiology, Physiology, Microbial communities

## Abstract

Sleep restriction alters gut microbiota composition and intestinal barrier function in rodents, but whether similar effects occur in humans is unclear. This study aimed to determine the effects of severe, short-term sleep restriction on gut microbiota composition and intestinal permeability in healthy adults. Fecal microbiota composition, measured by 16S rRNA sequencing, and intestinal permeability were measured in 19 healthy men (mean ± SD; BMI 24.4 ± 2.3 kg/m^2^, 20 ± 2 years) undergoing three consecutive nights of adequate sleep (AS; 7–9 h sleep/night) and restricted sleep (SR; 2 h sleep/night) in random order with controlled diet and physical activity. α-diversity measured by amplicon sequencing variant (ASV) richness was 21% lower during SR compared to AS (*P* = 0.03), but α-diversity measured by Shannon and Simpson indexes did not differ between conditions. Relative abundance of a single ASV within the family Ruminococcaceae was the only differentially abundant taxon (*q* = 0.20). No between-condition differences in intestinal permeability or β-diversity were observed. Findings indicated that severe, short-term sleep restriction reduced richness of the gut microbiota but otherwise minimally impacted community composition and did not affect intestinal permeability in healthy young men.

## Introduction

The gut microbiota is increasingly implicated as a possible mediator of the diverse acute and chronic adverse health effects associated with inadequate or disrupted sleep^1,2^. In rodents, sleep deprivation and sleep fragmentation alter gut microbiota composition, and induce inflammation, gut barrier damage and intestinal permeability^3–10^. Those effects are thought to be initiated, in part, by hypothalamic–pituitary–adrenal (HPA) axis activation, to be interrelated and bidirectional, and to contribute to metabolic and physiologic disturbances resulting from inadequate or disrupted sleep^1,2^.

The extent to which similar effects occur in humans is unclear. In one recent study, circulating markers of HPA-axis activation, inflammation and intestinal permeability were increased and gut microbiota composition was altered following 40 h total sleep deprivation in healthy adults^11^. Importantly in that same study, transplantation of the sleep-deprived human gut microbiota into germ free recipient mice resulted in intestinal permeability, inflammation as measured by circulating cytokine concentrations and cognitive impairment^11^. However, other studies have reported minimal or no effects of restricting sleep opportunities to 4 h/night for two to five consecutive nights on human gut microbiota composition^12,13^. These inconsistencies may be attributable to differences in study populations and design or may suggest that effects of sleep restriction on the human gut microbiota vary by the magnitude and duration of sleep restriction. Additional studies employing novel sleep restriction paradigms are therefore needed to increase understanding of relationships between sleep duration, gut microbiota composition and intestinal barrier function.

Severe (< 4 h/night), short-term (< 7 days) sleep deprivation is one of several occupational stressors commonly experienced by certain populations such as military personnel, first responders and ultra-endurance athletes^14–16^. Our laboratory has reported changes in gut microbiota composition concomitant with increased intestinal permeability and inflammation in individuals exposed to environments consisting of multiple stressors that include sleep deprivation^17,18^. This study was conceived to isolate the potential role of sleep deprivation in those results and extend the evidence base by determining the effects of severe (2 h sleep opportunity/night), short-term (3 consecutive nights) sleep deprivation on gut microbiota composition and intestinal permeability in healthy adults.

## Methods

### Participants and study design

This report details a sub-study included in a parent trial (clincaltrials.gov #NCT03525184) designed to test the efficacy of a multi-nutrient nutrition intervention for mitigating sleep deprivation-induced decrements in immune function^19,20^. Healthy men and women, 17–45 years with BMI < 30 kg/m^2^, who regularly slept 7–9 h/night, had not used oral antibiotics in the previous three months, and had no history of gastrointestinal disease, cardiometabolic disease or neurologic disorders were recruited from the Natick Soldier Systems Center, Natick, MA and surrounding area between February 2018 and April 2019. Consuming dietary supplements and probiotic-containing foods was prohibited for 2-weeks prior and throughout the study. The study was approved by the Headquarters U.S. Army Medical Research and Development Command Institutional Review Board, investigators adhered to the policies regarding protection of human subjects as prescribed in Army Regulation 70-25, the research was conducted in adherence with the provisions of 32 CFR Part 219, and all volunteers gave their free and informed consent to participate.

**Figure 1:**
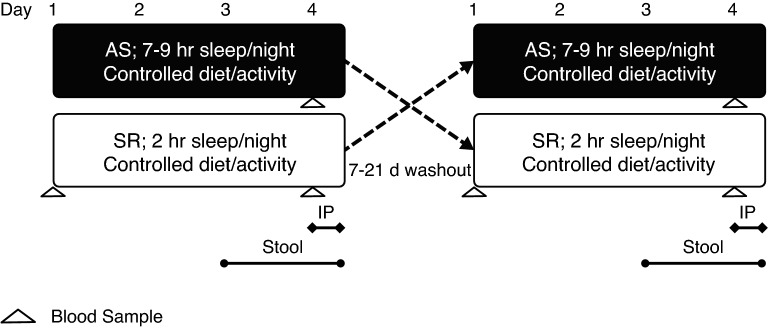
Study design. Randomized, crossover study in which participants completed three consecutive nights of adequate sleep (AS) or sleep restriction (SR). Stool sample collection began after waking on day 3 and continued until a sample was collected. Intestinal permeability (IP) measurements were conducted over 5 h on the morning of day 4.

This sub-study used a randomized, crossover design consisting of two, 3-day conditions (Fig. 1): AS (7–9 h sleep opportunity/night) and SR (2 h sleep opportunity/night). Order of completion was randomized using computer-generated randomization and phases were separated by a 7-day washout period when AS preceded SR and a 21-day washout period when SR preceded AS. During SR, participants reported to the laboratory the evening before sleep restriction began and were given 8.5 h time in bed to sleep that night. Thereafter, participants were under constant staff supervision for 24 h/day, and only provided 2 h/night (0430–0630) sleep opportunity for three consecutive nights. During AS, participants stayed in the research laboratory under staff supervision from 0630 to 2000 for three consecutive days and were instructed to sleep 7–9 h/night at their residence. Adherence to sleep instructions were monitored using self-report and wrist-worn actigraphy monitors (Actical, Philips Respironics, Murrysville, PA, USA) that measured total sleep time and time in bed. Herein, “sleep opportunity” refers to time in bed. Full study methods are reported elsewhere^19,20^ but briefly summarized below for context.

During both phases participants were provided a measured diet designed by Registered Dietitians to maintain energy balance (energy intake = energy expenditure). The diet was comprised of commercially available food items and designed to contain 0.9 g protein/kg body weight/day, to provide 65 ± 2% kcal from carbohydrate and 25 ± 2% kcal from fat, and to meet recommended intakes for omega-3 fatty acids, calcium, zinc, and vitamins A, C, and D. Participants were required to consume all provided foods and beverages, consumption was monitored and weighed by trained study staff, and no other foods or beverages other than water were allowed.

Participants also completed low-intensity prescribed exercise (walking and light cycle ergometry) during both phases. Total daily energy expenditure was estimated by determining the amount of time sleeping, participating in activities of daily living and study activities (e.g., playing video games, hygiene, etc.), and engaged in prescribed exercise^19,20^. Metabolic equivalents (METS) were assigned to each activity and used to calculate energy expenditure from time spent in each activity. The calculation used 0.9 METS to estimate energy expenditure during sleep, 1.25 METS to estimate energy expenditure while awake and engaged in activities of daily living, 2.5 METS to estimate energy expenditure during a marksmanship test administered during SR only, and activity-specific METs for all prescribed exercise^21^. Therefore, planned total energy expenditure was higher during SR than AS, and the increase was ~ 0.35 METS over 6 h (i.e., planned difference in total sleep time/night between AS and SR).

### Stool sample collection and analysis

The first stool sample produced after waking on the third day of each phase (i.e., after 48 h of adequate sleep or sleep restriction) was collected to assess microbiota composition. The average clock time of sample collection was similar between conditions (AS = 1255 [range: 0600–1900], SR = 1357 [range: 0005–2335]), and the amount of time elapsed between sample collection and processing was ≤ 60 min for all but two samples collected during SR (median [IQR]: AS = 10 min [15 min]; SR = 10 min [25 min], *P* = 0.29). Samples were collected in plastic containers and immediately refrigerated. Stool was assessed for consistency by study staff using the Bristol Stool Scale, and then processed and frozen at − 80 °C until analysis.

DNA extraction was conducted using the QIAamp Power Fecal DNA Kit (Qiagen, Inc., Hilden, Germany) according to manufacturer protocol. Extracted DNA was quantified using the NanoDrop 2000 spectrophotometers (Thermo Fisher Scientific, Waltham, MA, USA) and all samples passed the quality cut-off at DIN > 8. Amplicon sequencing of the 16S rRNA gene was conducted following the Illumina 16S ribosomal DNA Metagenomics Library Preparation manual (Illumina, Inc., San Diego, CA, USA) according to our established protocol^22^. Briefly, vendor recommended sets of primers isolated the hyper-variable V3 and V4 regions of the 16S rRNA amplicon, samples were barcoded, and amplicons subsequently generated. The Illumina MiSeq platform was used to sequence the 300 bp paired-end reads in a single run. The end of each read was overlapped to generate high quality, full-length reads of the V3 and V4 regions. De-multiplexed sequences underwent data quality assessment, processing, and chimera detection using QIIME2 v.2020.8^23^. Raw read sequences were joined using the q2-vsearch plugin, followed by initial quality filtering based on the quality scores using q2-demux and denoising with the DADA2^24^ plugin. All amplicon sequence variants (ASV) were aligned with MAFFT^25^ via q2‐alignment and used to construct a phylogeny with FastTree2 via q2-phylogeny^26^. To assign taxonomy, a Naïve Bayes Classifier was trained on the 16S rRNA V3–V4 region with the specific primers and the Greengenes^27^ v13.8 99% operational taxonomic unit database of reference sequences using q2-feature-classifier via classify-sklearn.

### Intestinal permeability

Intestinal permeability was assessed using a dual sugar absorption test administered at 0715 on the 4th day of each phase (i.e., after 72 h of adequate sleep or sleep deprivation). Participants consumed a beverage containing 5 g lactulose and 4 g mannitol dissolved in 180 mL of water, and then collected all urine produced over the next 5 h. Participants were sedentary throughout the urine collection period and consumed a standardized meal at 0800 to assess outcomes unrelated to this report^20^. Aliquots of the 5-h collection were immediately frozen and stored at − 80 °C.

Urine lactulose and mannitol concentrations were measured by HPLC (Agilent 1100 HPLC, Santa Clara, CA, USA) as previously described^28^. Fractional excretion of each probe was calculated by multiplying the measured concentration by the total volume of urine collected and dividing by the dose administered. The ratio of the fractional excretions of lactulose and mannitol from 0 to 5 h predominantly reflects small intestinal permeability though colonic permeability may also be captured in some cases due to inter-individual variability in transit time^29^. Urine lactulose concentrations were below the lower detectable limit of the assay for 50% of samples, which did not differ by condition (NS = 53%, SR = 47%; *P* = 1.0). For those samples, lactulose concentrations were set to ½ of the lower detectable limit prior to analysis. Additionally, two participants who did not consume the lactulose- and mannitol-containing beverage during AS were excluded from intestinal permeability analyses.

### Blood biochemistries

Fasted blood samples were collected at 0700–0715 on the morning of AS day 4 and SR days 1 and 4 by antecubital venipuncture. Serum was separated and stored at − 80 °C until analysis. Serum high sensitivity C-reactive protein (hsCRP) concentrations were measured using the Luminex^®^ multiplex platform (MAGPIX System; Luminex, Austin, TX, USA) with xPONENT^®^ software (EMD Millipore, Burlington, MA, USA). Serum cortisol concentrations were measured using the Immulite 2000 immunoassay system (Siemens Healthcare, Erlangen, Germany).

### Statistical analysis

Sample size estimates were based on means and standard deviations measured in our previous work^18^ and indicated that 15 participants would allow detection of a 55% increase in intestinal permeability at power = 80% and alpha = 0.05. Between-condition differences in dietary intake and within-condition differences in serum biomarkers were assessed by paired *t* test. Between-condition differences in serum biomarkers, intestinal permeability and α-diversity (observed ASVs, Shannon index, Simpson index) were assessed by linear mixed models with condition (AS or SR), sequence order (AS then SR or SR then AS) and study phase included as fixed factors and subject included as a random intercept. Normal distribution of residuals and heterogeneity of variance were examined to verify adherence to model assumptions and log_10_-transformations were used if needed to meet model assumptions. No carryover effects were observed.

Between-condition differences in gut microbiota community composition were analyzed by PERMANOVA (999 permutations) using the Adonis plugin in QIIME2 v.2020.8. Differential abundance analyses were completed at the phyla, genus and ASV-levels using Microbiome Multivariable Associations with Linear Models (MaAsLin 2)^30^. Differential abundance analyses included taxa detected in ≥ 25% of samples, used a linear mixed effects model with total sum scaling normalization and log transformation, controlled for individual effects, and included condition, sequence order and study period as fixed factors. False discovery rate (*q-*value) was controlled using the Benjamini–Hochberg correction. Associations between outcomes were assessed using Pearson’s or Spearman’s rank correlation as appropriate. Analyses were completed in SPSS v.21 (IBM, Armonk, NY, USA) and R v. 4.0.3. Statistical significance was defined as *P* ≤ 0.05 and *q* ≤ 0.20. Data are presented as mean (SD) or median [IQR] unless otherwise noted.

## Results

Twenty-four men were randomized in blocks of two to four participants (AS then SR, n = 10; SR then AS, n = 14). Three participants withdrew for personal reasons, one was withdrawn for not meeting an eligibility requirement, and another was withdrawn due to an unrelated medical condition leaving 19 participants for analysis (AS then SR, n = 8; SR then AS, n = 11).

Participants self-reported an average weekday wake time of 0537 [range: 0430–0610], which was consistent with actigraphy data collected the weekdays preceding SR (0546 [range: 0451–0655]) and during AS (0533 [range: 0446–0600]). Actigraphy data indicated participants slept 125 ± 12 min/night during SR and 449 ± 51 min/night during AS. Mean energy intake was slightly higher during SR compared to AS (mean difference = 115 kcal/day [95% CI 67, 164], *P* < 0.001) to account for differences in energy expenditure resulting from less time asleep during SR; however, calculated energy balance did not differ between conditions Supplementary Table S1). Mean body weight increased 0.5 kg ([95% CI 0.2, 0.8], *P* = 0.005) from day 1 to day 2 of SR but was stable thereafter and did not change over time during AS (Supplementary Table S2). No participant reported consuming any foods or beverages other than those provided by study staff during SR and AS.

### Stress, inflammation and intestinal permeability

**Figure 2:**
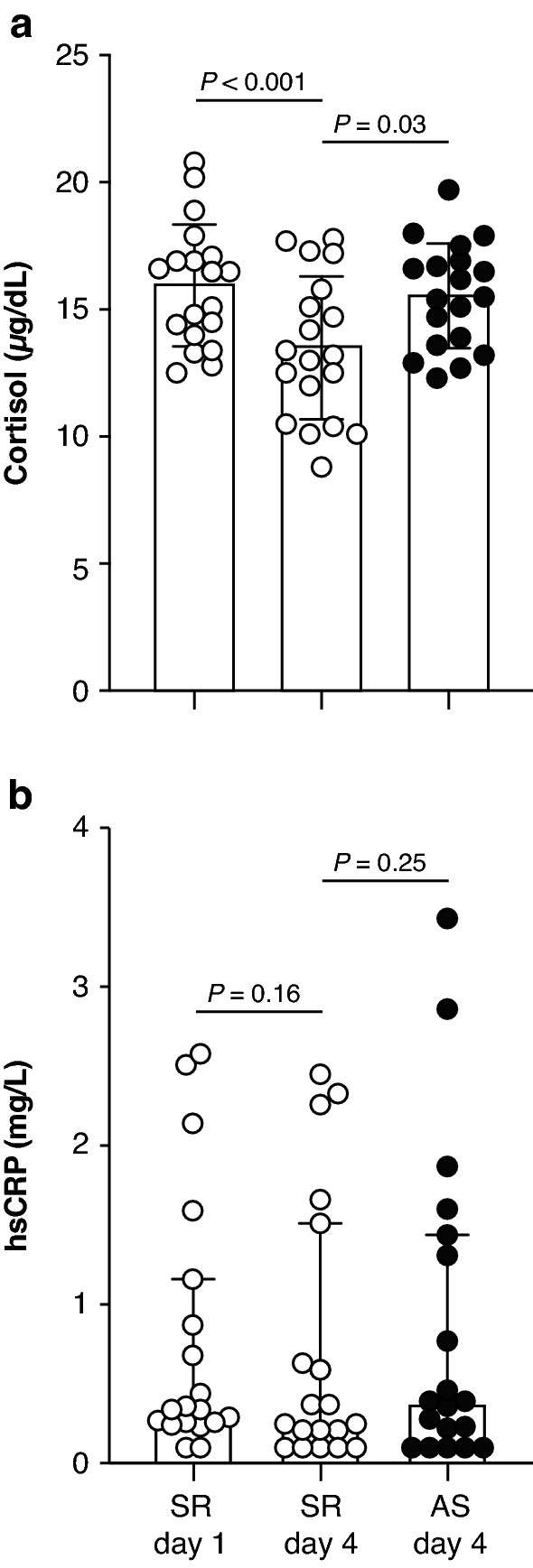
Serum markers of stress and inflammation following three consecutive nights of adequate sleep (AS) or sleep restriction (SR). (**a**) Serum cortisol, Mean ± SD shown. (**b**) High sensitivity C-reactive protein (hsCRP; log_10_-transformed for analysis). Median and interquartile range shown. (**a**,**b**) Within-condition comparisons analyzed by paired *t* tests. Between-condition comparisons analyzed by mixed model ANOVA. n = 19.

Serum cortisol concentrations decreased from SR day 1 to SR day 4 and were lower on SR day 4 relative to AS day 4 (Fig. 2a and Supplementary Table S3). Serum hsCRP concentrations did not differ between SR day 1 and SR day 4 or between SR day 4 and AS day 4 (Fig. 2b and Supplementary Table S3).

**Figure 3:**
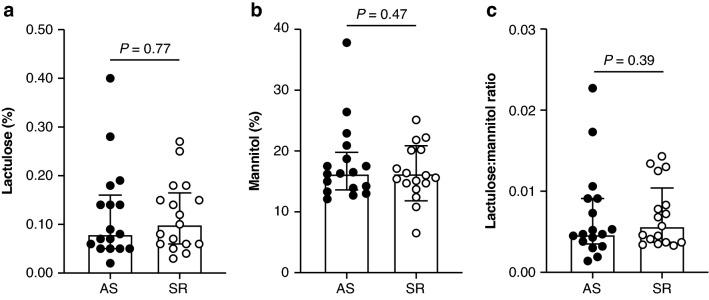
Intestinal permeability measured by urinary excretion of sugar substitutes following three consecutive nights of adequate sleep (AS) or sleep restriction (SR). Five-hour urinary excretion of (**a**) lactulose and (**b**) mannitol expressed as percent of dose administered. (**c**) Lactulose:mannitol ratio. (**a**–**c**) Mixed model ANOVA; all data log_10_-transformed for analysis. Median and interquartile range shown. n = 17.

Total urine volume did not differ by condition (Supplementary Table S4). No between-condition differences were observed for lactulose or mannitol excretion, or the lactulose:mannitol ratio (Fig. 3a–c and Supplementary Table S4). Excluding 11 participants with a lactulose concentration below the detectable limit of the assay during one or more conditions did not change those results (*P* > 0.19 for all).

### Gut microbiota composition

**Figure 4:**
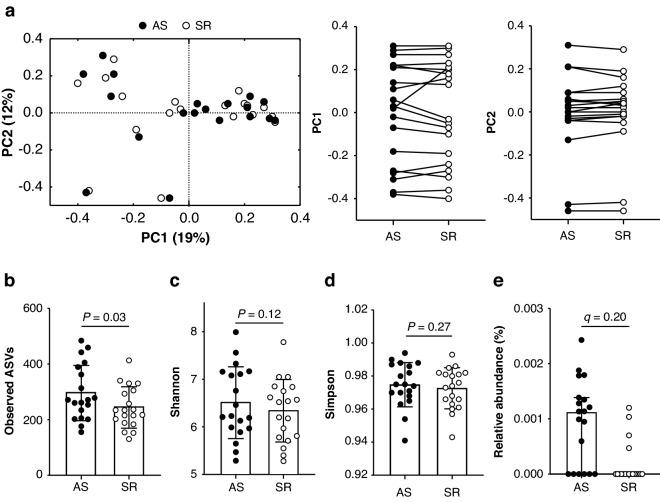
Gut microbiota composition following three consecutive nights of adequate sleep (AS) or sleep restriction (SR). (**a**) Principal coordinates analysis of Bray–Curtis dissimilarities (PERMANOVA, *P* = 1.0). (**b**–**d**) Between-condition differences in α-diversity analysed by mixed model ANOVA. Mean ± SD shown. (**e**) Differential abundance analyses of phyla, genera and amplicon sequencing variants (ASVs) by Microbiome Multivariable Associations with Linear Models identified ASV_Firmicutes.Clostridia.Clostridiales.Ruminococcaceae as the only differentially abundant taxon (*P* ≤ 0.05; *q* ≤ 0.20). Median and interquartile range shown. n = 19.

Stool consistency did not differ between conditions (AS = 3 [2]; SR = 3 [1.5], *P* = 0.21). A median of 39,195 [range = 9771–108,984] reads were obtained from stool samples, which did not differ by condition (AS = 35,150 [19,686]; SR = 32,963 [23,670], *P* = 0.28). Reads were assigned to 3275 unique ASVs comprising 12 phyla and 98 genera. Principal coordinates analysis of Bray–Curtis (Fig. 4a), weighted UniFrac, and unweighted UniFrac distances (Supplementary Fig. S1) did not indicate measurable shifts in community composition due to sleep restriction (PERMANOVA, P = 1.0 for all). α-diversity measured by observed ASVs was 21% lower during SR relative to during AS while no between-condition differences in Shannon or Simpson diversity indexes were observed (Fig. 4b–d), indicating that community richness was reduced by sleep restriction, but evenness was not affected.

In differential abundance analyses, nine ASVs, three genera and no phyla demonstrated significant between-condition differences in relative abundance (*P* < 0.05, Supplementary Table S5). However, only one ASV within the family Ruminococcaceae (Fig. 4e) remained significantly different after false discovery rate adjustment (*P* = 0.001; *q* = 0.20). That result suggested that the lower community richness observed during SR was attributable to a loss of rare taxa given that ASVs detected in < 25% of samples were excluded from differential abundance analyses.

### Correlations

The between-condition difference in serum cortisol concentrations was correlated with the corresponding difference in lactulose:mannitol ratio (ρ = 0.55, *P* = 0.02). No additional correlations were observed among the between-condition differences in cortisol, hsCRP, lactulose:mannitol ratio or α-diversity metrics (*P* > 0.05). Between-condition differences in cortisol, hsCRP, and lactulose:mannitol ratio were also not correlated with between-condition differences in the nine ASVs and three genera identified in differential abundance analyses (*P* > 0.05).

## Discussion

The extent to which sleep restriction alters human gut microbiota composition and intestinal barrier function is unclear as few studies exist and findings of those studies are inconsistent. The present study extends the evidence base by demonstrating that 2 h sleep opportunity/night over 2–3 consecutive nights reduces gut microbiota community richness without affecting relative abundances of prevalent taxa or intestinal permeability.

The observed reduction in gut microbiota community richness in the absence of differences in other α-diversity metrics, β-diversity and taxa relative abundances both extends and contrasts with previous reports. In one recent study, alterations in β-diversity, reduced gut microbiota community richness but not evenness, and changes in the relative abundances of several genera were reported following 40 h of total sleep deprivation in healthy adults^11^. In other studies, restricting sleep opportunities to 4 h/night over 2 or 5 consecutive nights had no effect on any diversity metrics and few, if any, effects on taxa relative abundances^12,13^. Reasons for the inconsistencies are unclear and results should be interpreted cautiously given small cohort sizes (n = 9–25), missing data and varying levels of control for potentially confounding factors such as dietary intake and whether bed and wake times were delayed or advanced. Alternately, study results may collectively suggest that effects of short-term sleep restriction on the human gut microbiota progressively increase with the severity of sleep restriction such that effects are most pronounced following total sleep deprivation. Further, a reduction in community richness appears to be emerging as a characteristic of severe sleep restriction, and based on the present results, may be driven by the loss of rare taxa. This is potentially concerning for populations in which severe sleep restriction is repeated and common, given that extinctions of taxa reduce the functional repertoire of the gut microbiota and are not easily reversed^31^, and higher diversity is generally associated with community resilience and better host health^32^.

Intestinal permeability was unaffected by the sleep restriction paradigm imposed herein. That result is consistent with the absence of increases in circulating cortisol and lack of between-condition differences in hsCRP concentrations given that stress-induced increases in intestinal permeability can promote inflammation by allowing translocation of antigens from the intestinal lumen into circulation^33^. Of note, previous studies have collectively failed to demonstrate increases in circulating CRP following short-term partial or total sleep deprivation; whereas, increases are observed with chronic inadequate sleep^34^. In contrast, multiple circulating biomarkers of intestinal barrier dysfunction, stress (cortisol) and inflammation (not including hsCRP) were increased concomitant to changes in gut microbiota composition in healthy adults following 40 h of total sleep deprivation^11^. In that same study, fecal microbiota transplantation experiments provided evidence that the intestinal barrier dysfunction observed was mediated by sleep deprivation-induced changes in the gut microbiota and contributed to systemic inflammation, blood brain barrier permeability and cognitive deficits^30^. Results of the present study may therefore suggest that as few as 2 h sleep opportunity/night is sufficient to block activation of the same pathways, at least over the 72-h time period studied.

Inconsistencies in gut microbiota, intestinal barrier and inflammation responses across studies may also be attributable, in part, to differences in methods and outcomes used to measure intestinal barrier function and inflammation. For example, the dual-sugar absorption test and 5-h urine collection period used in the present study does not assess large intestinal permeability^29^ or the immunological component of intestinal barrier function^33^. In contrast, the circulating biomarkers measured by Wang et al.^11^ during 40-h of sleep deprivation collectively capture both physical and immunological components of barrier function throughout the entire gastrointestinal tract^35^. Results also differ from the collective findings of multiple rodent studies wherein sleep deprivation and sleep fragmentation have induced potentially unfavourable changes in gut microbiota composition, decrements in intestinal barrier function and inflammation^4–9^. However, extrapolating those findings to humans is tenuous given that in rodent studies experimental durations are often longer^1^, methods of preventing sleep often exacerbate HPA-axis activation^36^ which could directly impact the gut microbiota and intestinal barrier^37^, coprophagy is rarely prevented^38^, and levels of control over diet and physical activity vary^1,2^. Additionally, timing of stool sample collections may influence gut microbiota composition^39^. The inability to standardized collection timing in human studies may therefore add variability that could mask effects seen in pre-clinical studies where collection timing can be standardized. Nonetheless, several rodent studies have used fecal microbiota transplantation^6,11^, germ free models^11^, vagotomy^40^ and culturing of peripheral tissues^4^ to implicate the gut microbiota as a potential mediator of sleep restriction-induced decrements in intestinal barrier function, inflammation and host health. As such, there remains a need for additional studies employing different sleep restriction paradigms to fully elucidate the link between sleep, the human gut microbiota and host physiology.

Study strengths include the randomized crossover design and tight control of diet and activity during the AS and SR periods. This approach overcomes some limitations of previous studies that have relied on longitudinal study designs^11,12^ or have not controlled dietary intake^12^, either of which may introduce factors that impact the gut microbiota independent of sleep restriction. A limitation of this study includes the unexplained but small increase in body weight from day 1 to 2 of SR. However, any impact is likely minimal as body weights were stable after day 1 during both conditions and estimated energy balances did not differ. Additional study limitations include allowing volunteers to sleep at their residence during AS. However, during AS participants were supervised most of the day, did not report consuming foods or beverages outside of the laboratory, adhered to the sleep prescription of 7–9 h/night based on actigraphy data, and the approach ensured participants slept in a comfortable and familiar setting. An additional limitation is reliance on 16S rRNA amplicon sequencing given that this approach cannot measure outcomes such as changes in functional capacity and metabolic output of the gut microbiota, which may precede shifts in community composition, and would provide additional insight into the community’s role in mediating effects of sleep restriction on host physiology and health outcomes. Results also should be interpreted within the context of the sleep restriction paradigm imposed, and may not extend to longer periods of sleep restriction including chronic inadequate sleep, or reflect effects of sleep fragmentation and circadian misalignment, all of which may have unique effects on the gut microbiota and intestinal physiology^1,2^. Finally, results may not be generalizable to women, and future studies should seek to investigate sex differences in any effects of sleep restriction on the gut microbiota.

In summary, the severe, short-term sleep restriction paradigm studied herein, which is applicable to certain populations such as military personnel and first responders, reduced gut microbiota community richness but otherwise had little measurable impact on gut microbiota composition or intestinal permeability. When interpreted in the context the current evidence base, these findings suggest that interactions between sleep, the gut microbiota and host physiology are likely to vary according to the magnitude and duration of sleep disruption imposed and underscore a need for additional translational research.

## Supplementary Information

Supplementary Information.

## Data Availability

The data supporting study findings are available from the corresponding author upon reasonable request and pending ethical and legal approvals. The data are not publicly available due to ethical restrictions.
