# Supplementary material for: Severe, short-term sleep restriction reduces gut microbiota community richness but does not alter intestinal permeability in healthy young men
Source: Sci Rep. 2023 Jan 5;13:213. doi: 10.1038/s41598-023-27463-0 (PMC9816096; doi:10.1038/s41598-023-27463-0)
Supplement: Supplementary file 1 — Supplementary Information. [file 41598_2023_27463_MOESM1_ESM.pdf]

Supplementary Material

Severe, short-term sleep restriction reduces gut microbiota community richness but does not alter intestinal permeability in healthy young men

J. Philip Karl[1]*, Claire C. Whitney[1], Marques A. Wilson[1], Heather S. Fagnant[1], Patrick N. Radcliffe[1,2], Nabarun Chakraborty[3], Ross Campbell[4], Allison Hoke[3], Aarti Gautam[3], Rasha Hammamieh[3] and Tracey J. Smith[1]

[1]U.S. Army Research Institute of Environmental Medicine, Natick, MA, USA
[2]Oak Ridge Institute of Science and Education, Oak Ridge, TN, USA
[3]Medical Readiness Systems Biology, CMPN, Walter Reed Army Institute of Research, Silver Spring, MD, USA
[4]Geneva Foundation, Walter Reed Army Institute of Research, Silver Spring, MD, USA.

***Corresponding author:**
J. Philip Karl
US Army Research Institute of Environmental Medicine
Military Nutrition Division
10 General Greene Ave; Bldg 42
Natick, MA 01760
james.p.karl.civ@health.mil
+01 508-206-2318

Supplementary Material

Supplementary Table S1. Energy and macronutrient intakes and energy balance during three consecutive days of adequate sleep (AS) or sleep restriction (SR).

	**AS (mean ± SD)**	**SR (mean ± SD)**	**Mean difference [95% CI]**	***P*-value†**
Energy (kcal/d)	2762 ± 349	2877 ± 339	115 [67, 164]	<0.001
Carbohydrate (g/d)	432 ± 64	459 ± 64	27 [18, 35]	<0.001
Fat (g/d)	84 ± 10	85 ± 8	1 [-2, 3]	0.55
Protein (g/d)	68 ± 6	69 ± 7	1 [-1, 2]	0.36
Energy Balance (kcal/d)	73 ± 131	42 ± 106	-31 [-72, 10]	0.12

n = 19.
†Paired samples *t*-test.

Supplementary Material

Supplementary Table S2. Morning fasted body weights measured during three consecutive days of adequate sleep (AS) or sleep restriction (SR).

	Day 1	Day 2	Day 3	Day 4	*P*–value[†]		
					**Condition**	**Day**	**Condition* Day**
**AS**	77.0 ± 7.2	76.9 ± 7.4	76.9 ± 7.4	76.9 ± 7.5	<0.001	0.71	0.22
**SR**	75.9 ± 7.6[a]	76.4 ± 7.4[b]	76.3 ± 7.3[b]	76.4 ± 7.6[b]			

[†]Linear mixed model with condition, day and their interaction as fixed factors and subject as a random intercept.
[a,b]Linear mixed model within condition, including day as a fixed factor and subject as a random intercept. Main effect of day within AS, $P = 0.84$. Main effect of day within SR, $P = 0.02$. Within a row, values not sharing a superscript letter are significantly different, $P < 0.05$.

Supplementary Material

Supplementary Table S3. Serum markers of stress and inflammation following three consecutive nights of adequate sleep (AS) or sleep restriction (SR).

	**SR-day 1**	**SR-day 4**†	**AS-day 4**‡
Cortisol (µg/dL; mean ± SD)	16.0 ± 2.4	13.5 ± 2.8***	15.5 ± 2.1*
hsCRP (mg/L; median [IQR])§	0.34 [0.90]	0.25 [1.41]	0.39 [1.34]

n = 19.
†Within-condition comparisons analyzed by paired *t*-tests. ***Significantly different from SR-day 1 ($P < 0.001$).
‡Between-condition comparisons analyzed by mixed model ANOVA. *Significantly different from SR-day 4 ($P = 0.03$).
§Log_{10}-transformed for analysis.

Supplementary Material

Supplementary Table S4. Intestinal permeability measured following three consecutive nights of adequate sleep (AS) or sleep restriction (SR).

	**AS**	**SR**	***P*-value**[†]
Urine volume (mL; mean ± SD)	985 ± 359	1063 ± 249	0.53
Lactulose (%; median [IQR])[‡]	0.08 [0.10]	0.10 [0.11]	0.77
Mannitol (%; median [IQR])[‡]	16.3 [6.2]	15.6 [5.9]	0.47
Lactulose:mannitol ratio (median [IQR])[‡]	0.0047 [0.0056]	0.0057 [0.0067]	0.39

n=17.
[†]Mixed model ANOVA.
[‡]Log_{10}-transformed for analysis.

Supplementary Material

Supplementary Table S5. Genera and amplicon sequencing variants (ASVs) demonstrating between-condition differences ($P < 0.05$) following two consecutive days of adequate sleep (AS) or sleep restriction (SR).[†]

**Taxon**	**β ± SE**[‡]	***P*-value**	***q*-value**
Genera			
Campylobacter	-0.32 ± 0.10	0.007	0.29
Roseburia	-0.17 ± 0.07	0.02	0.43
Papillibacter	0.26 ± 0.12	0.04	0.57
ASV			
Firmicutes.Clostridia.Clostridiales.Ruminococcaceae	-0.39 ± 0.10	0.001	0.20
Firmicutes.Clostridia.Clostridiales.Lachnospiraceae.	-0.34 ± 0.13	0.02	0.92
Clostridia.Clostridiales.Lachnospiraceae.*Clostridium*	-0.26 ± 0.10	0.02	0.92
Firmicutes.Clostridia.Clostridiales.Ruminococcaceae. *Subdoligranulum variabile*	-0.30 ± 0.11	0.02	0.92
Firmicutes.Clostridia.Clostridiales.Ruminococcaceae	-0.19 ± 0.08	0.03	0.92
Firmicutes.Clostridia.Clostridiales.Ruminococcaceae	-0.31 ± 0.13	0.03	0.92
Bacteroidetes.Bacteroidia.Bacteroidales.Bacteroidaceae. *Bacteroides*	0.25 ± 0.11	0.03	0.92
Bacteroidetes.Bacteroidia.Bacteroidales.Prevotellaceae. *Prevotella*	-0.12 ± 0.06	0.04	0.92
Firmicutes.Clostridia.Clostridiales.Lachnospiraceae. *Roseburia inulinivorans*	-0.14 ± 0.07	0.05	0.92

[†]Analyzed by Microbiome Multivariable Associations with Linear Models[1] with treatment, treatment order and study period included as fixed factors, and subject as a random factor.
[‡]AS is the reference group.

[1]Mallick H, Rahnavard A, McIver LJ, et al. Multivariable association discovery in population-scale meta-omics studies. *PLoS Comput Biol.* 2021;17(11):e1009442.

Supplementary Material

Supplemental Figure 1

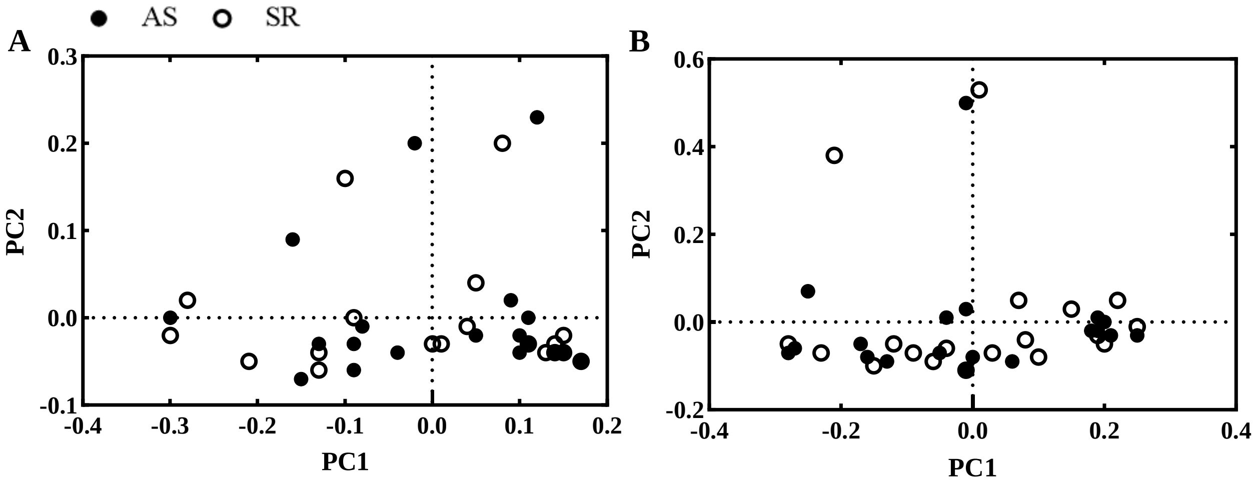

Supplemental Figure 1. Gut microbiota composition following two consecutive days of adequate sleep (AS) or sleep restriction (SR). Principal coordinates analysis of **(A)** weighted and **(B)** unweighted Unifrac distances. PERMANOVA, P = 1.0.
